# Design of Three-Dimensional Scaffolds with Tunable Matrix Stiffness for Directing Stem Cell Lineage Specification: An In Silico Study

**DOI:** 10.3390/bioengineering4030066

**Published:** 2017-07-27

**Authors:** Sanjairaj Vijayavenkataraman, Zhang Shuo, Jerry Y. H. Fuh, Wen Feng Lu

**Affiliations:** 1 Department of Mechanical Engineering, National University of Singapore (NUS), Singapore 117576, Singapore; fred1995insg@gmail.com (Z.S.); jerry.fuh@nus.edu.sg (J.Y.H.F.); mpelwf@nus.edu.sg (W.F.L.); 2 NUS Research Institute, Suzhou Industry Park, Suzhou 215123, China

**Keywords:** 3D scaffolds, scaffold design, tissue engineering, regenerative medicine, stem cells

## Abstract

Tissue engineering is a multi-disciplinary area of research bringing together the fields of engineering and life sciences with the aim of fabricating tissue constructs aiding in the regeneration of damaged tissues and organs. Scaffolds play a key role in tissue engineering, acting as the templates for tissue regeneration and guiding the growth of new tissue. The use of stem cells in tissue engineering and regenerative medicine becomes indispensable, especially for applications involving successful long-term restoration of continuously self-renewing tissues, such as skin. The differentiation of stem cells is controlled by a number of cues, of which the nature of the substrate and its innate stiffness plays a vital role in stem cell fate determination. By tuning the substrate stiffness, the differentiation of stem cells can be directed to the desired lineage. Many studies on the effect of substrate stiffness on stem cell differentiation has been reported, but most of those studies are conducted with two-dimensional (2D) substrates. However, the native in vivo tissue microenvironment is three-dimensional (3D) and life science researchers are moving towards 3D cell cultures. Porous 3D scaffolds are widely used by the researchers for 3D cell culture and the properties of such scaffolds affects the cell attachment, proliferation, and differentiation. To this end, the design of porous scaffolds directly influences the stem cell fate determination. There exists a need to have 3D scaffolds with tunable stiffness for directing the differentiation of stem cells into the desired lineage. Given the limited number of biomaterials with all the desired properties, the design of the scaffolds themselves could be used to tune the matrix stiffness. This paper is an in silico study, investigating the effect of various scaffold parameter, namely fiber width, porosity, number of unit cells per layer, number of layers, and material selection, on the matrix stiffness, thereby offering a guideline for design of porous tissue engineering scaffolds with tunable matrix stiffness for directing stem cell lineage specification.

## 1. Introduction

The term ‘tissue engineering’ was officially coined at a National Science Foundation workshop in 1988 to mean “the application of principles and methods of engineering and life sciences toward the fundamental understanding of structure-function relationships in normal and pathological mammalian tissues and the development of biological substitutes to restore, maintain, or improve tissue function” [1]. The field of tissue engineering aims to regenerate damaged tissues by combining cells from the body with highly-porous scaffold biomaterials, which act as templates for tissue regeneration, to guide the growth of new tissue. Far from being a passive component, scaffold material and porous architecture design play a significant role in tissue regeneration by preserving tissue volume, providing temporary mechanical function, and delivering biofactors [2]. Cell behavior is directly affected by the scaffold architecture since the extracellular matrix (ECM) provides cues that influence the specific integrin—ligand interactions between cells and the surrounding ECM [3]. Hence, the 3D scaffold environment can influence cell proliferation or direct cell differentiation. 

Stem cells have become almost synonymous with the term tissue engineering of late. Use of postnatal stem cells has the potential to alter the perspective of tissue engineering significantly. Successful long-term restoration of continuously self-renewing tissues, such as skin, for example, depends on the use of extensively self-renewing stem cells [4,5]. Mechanical properties of the substrate play a very important role in determining the differentiation of stem cells. Adult stem cells, as part of normal regenerative processes, are believed to egress and circulate away from their niche [6], and then engraft and differentiate within a range of tissue microenvironments. The tissue or matrix microenvironments can be as physically diverse as those of brain, muscle, and bone precursor osteoid [7]. In short, the scaffold properties, especially the stiffness, determine, to a large extent, the differentiation of stem cells into the neural lineage, muscle lineage, or bone lineage. There were detailed studies on how matrix stiffness governs the stem cell fate determination and the underlying mechanism [8,9,10]. Most of these studies are done on two-dimensional substrates. However, the in vivo tissue microenvironment is three-dimensional, and cells reside within a complex environment containing multiple ECM components, different cell types, and mixtures of cell-secreted factors. Hence, 3D cell culture models mimic the natural tissue environment more closely compared to traditional 2D cell culture. It is highly likely that, in 3D cultures, stem cell differentiation may be regulated differently, through the mechanical stiffness of the substrate. The matrix properties can be altered by using different materials, but one of the major challenges is the lack of suitable substrates with physiologically-relevant properties [8]. There exists a need to have 3D scaffolds with tunable stiffness for directing the differentiation of stem cells into the desired lineage. Given the limited number of biomaterials with all the desired properties, design of the scaffolds themselves could be used to tune the matrix stiffness. 

Most of the works published on designing of 3D porous scaffolds focus on the architecture of the scaffold, pore size, porosity, fiber diameter (in case of fibrous scaffolds), and the fabrication process [11,12]. The aims of the study were to design an in silico scaffold model with which to derive the effect of structural parameters on stiffness properties and inferred stem cell differentiation functionality. Taboas et al. [13] manufactured a Poly(lactic acid) (PLA) scaffold with complex internal architectures that mimicked human trabecular bone using indirect solid freeform fabrication (SFF). Similarly, Wilson et al. [14] have developed an indirect SFF method for ceramic scaffolds with defined and reproducible 3D porous architectures; they report ectopic bone formation for all scaffold and cell constructs. Sodian et al. [15] reported biocompatible and biodegradable scaffolds, made from thermoplastic elastomer poly-4-hydroxybutyrate (P4HB) and polyhydroxyoctanoate (PHOH), to be used for human pulmonary and aortic homografts. They have demonstrated that indirect SFF techniques are able to fabricate scaffolds with a physiological valve design and to mold a complete trileaflet heart valve without the need for suturing. Since the advent of additive manufacturing (or 3D printing) and bioprinting [16,17], these kinds of studies have gained much more attention. Other recent studies focused on maximizing the surface area of scaffolds with complex architecture [18,19,20] for fabrication of scaffolds that have heterogeneous internal structures with controlled porosity levels and architectures. 

However, there are only very few studies reported on the effect of 3D scaffold design on stem cell differentiation, though the phenomenon is studied experimentally in two-dimensional substrates [7,8,9]. In one study, Li et al. [21] fabricated photopolymerizable hydrogel scaffolds with different pore sizes using D-mannitol crystals as porogen particles and studied the differentiation of neural stem/progenitor cells (NSPCs) into neurons, astrocytes, and oligodendrocytes. Results indicated that larger pore sizes effectively promote NSPC 3D differentiation but, unlike 3D printing, the pore size and porosity could not be controlled precisely using this method, and it suffers from poor repeatability. Guvendiran et al. [22] fabricated scaffolds with square, hexagonal, and orthogonal pores using a pressure-activated microsyringe system. On culturing human mesenchymal stem cells (hMSCs), it was found that the percentage of osteogenic cells was significantly higher on scaffolds with hexagonal and octagonal patterns (~80%) as compared to those with square patterns (~65%). While these studies prove that the scaffold parameters influence stem cell differentiation, they only study the effect of one of the scaffold parameters on the differentiation of stem cells. There are other scaffold parameters that would affect the scaffold property and, hence, the differentiation, but not studied. Hence, there exists a gap in designing scaffolds with different matrix stiffness for stem cells fate determination, given the potential of stem cells in the field of tissue engineering.

This paper, as an in silico study, focuses on designing 3D porous scaffolds with tunable matrix stiffness for stem cell fate determination. Engler et al. [7] report the stiffness range for differentiation of stem cells into various lineages, namely neurogenic (0.1–1 kPa), myogenic (8–17 kPa), and osteogenic lineages (25–40 kPa and above). Various scaffold designs result in various stiffness values and the range suitable for neural, muscle, or bone cells can be appropriately chosen. To this end, the effect of fiber diameter, porosity, and pore size on the stiffness of the scaffold structure is studied first. Based on this understanding, the design is varied (number of unit cells in a layer, number of layers) to obtain scaffolds of varying stiffnesses, keeping the other parameters (pore size, porosity, fiber diameter) in a range preferred for cell culture. Additionally, the effect of using various materials on the stiffness of scaffolds with the same design is also studied.

## 2. Materials and Methods

**Figure 1:**
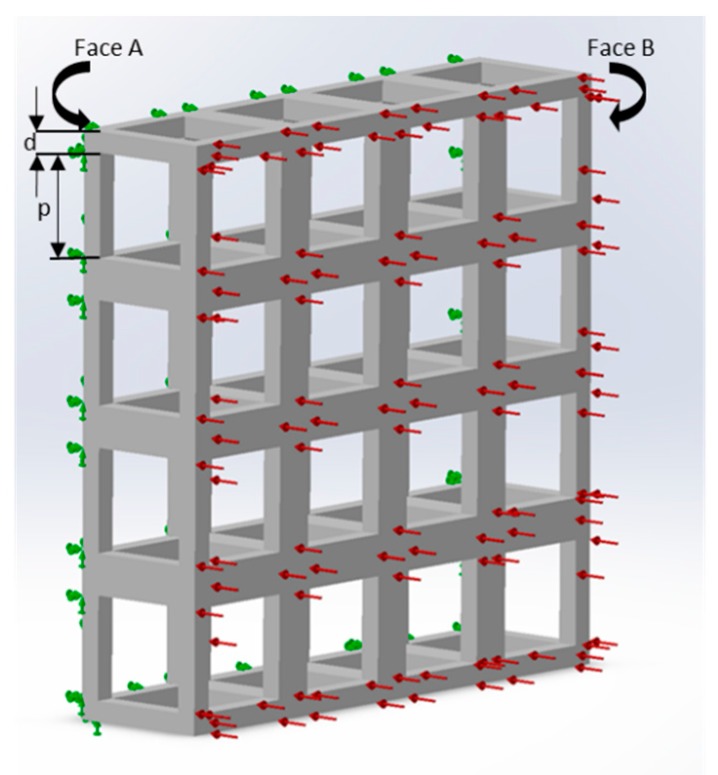
Loads and constraints for the numerical analysis of scaffolds under a tensile solicitation consisting of a single layer with 16 unit cells (G5, porosity = 80%, pore side length, *p* = 200 μm, and fiber width, *d* = 80 μm). Face A was constrained, and a uniform force in a single direction was imposed on Face B.

The current study used SolidWorks 2016 (Dassault Systèmes SOLIDWORKS Corp., Waltham, MA, USA) and ABAQUS (Dassault Systèmes SOLIDWORKS Corp., Waltham, MA, USA) software for modelling and analysis. For the simplicity of the study and ease of fabrication, only cubic unit cells were used. The meshing size was set at the ‘finest’ for all the structural analyses. Material properties of gelatin at three different concentrations (wt%) were considered for the purpose of simulation, namely 5% (G5), 7% (G7), and 14% (G14), with their elastic modulus (E_o_) of 40 kPa, 63 kPa, and 110 kPa, respectively, and a Poisson’s ratio of 0.4 [23]. In addition, other materials considered (in Section 4.4 and Section 4.5) were chitosan (E_o_ = 2.53 kPa and a Poisson’s ratio of 0.3) [24], and polycaprolactone (PCL) (E_o_ = 400 MPa and a Poisson’s ratio of 0.3) [19,25]. For the numerical computation of the elastic modulus (Figure 1), a uniform displacement in a single direction was considered (the *x* direction), which is equivalent to the strain on the same direction (ε_x_), imposed to a face of the block (Face B). The opposite face (Face A) of the scaffold unit was constrained and unable to have any displacement. The average reaction force produced on Face B was used to determine the elastic modulus, due to the imposed displacement. 

## 3. Equations

The following equations were used.

(1) $$\mathrm{Young’s}\;\mathrm{modulus},\;E=\frac{\sigma }{\epsilon }=\frac{\left(F/A\right)}{\left(\Delta L/L\right)}=\frac{P*L}{\Delta L}$$

where *E* is the Young’s modulus (elastic modulus), *F* is the applied force, σ is the stress, ϵ is the strain, *P* is the pressure, *A* is the area, *L* is the length, and ∆*L* is the change in length in the chosen direction, the value of which is obtained from the structural simulation. For the unit cell, ‘*p*’ is the pore side length, ‘*d*’ is the fiber width, and the unit cell side length (*L*) is given by *L* = *p* + (2 × *d*); for multiple cells along the chosen direction, ‘*L*’ should be the unit cell side length times the number of cells along the chosen direction. Since the unit cell is symmetric in the *x*, *y*, and *z* directions, the effective Young’s modulus is the same in the *x*, *y*, and *z* directions, i.e., *E = E_xx_ = E_yy_ = E_zz_* [11]. The elastic modulus is also referred to interchangeably as stiffness, as an accepted practice in the life sciences literature. 

Porosity is the ratio of volume of voids to volume of the structure without voids and given by the following equation [12]:

(2) $$Porosity=1-\left(\frac{Vsolid}{Vtotal}\right)*100$$

where *V_solid_* is the volume of the solid, and *V_total_* is the total volume of scaffold. 

## 4. Results

### 4.1. Influence of Fiber Width on the Stiffness of the Structure

**Figure 2:**
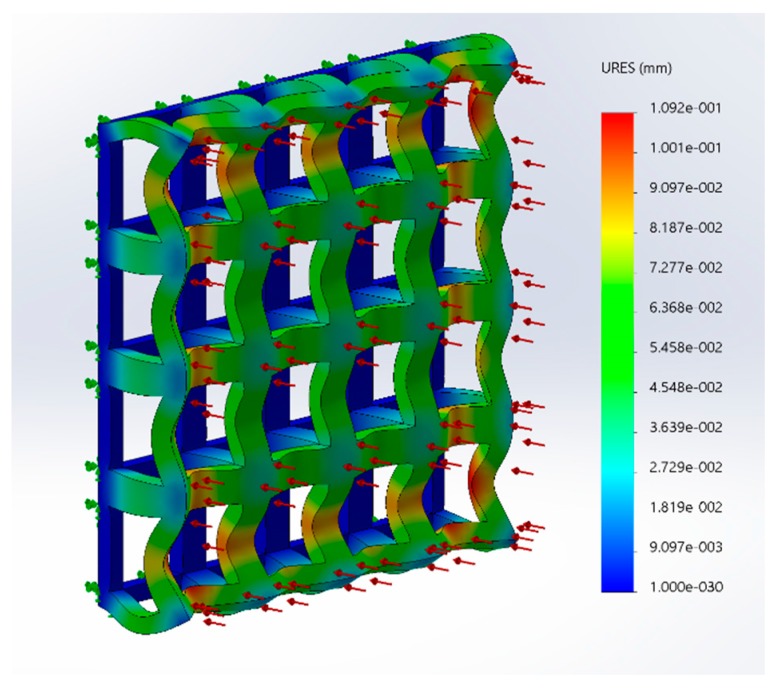
Structural simulation results of a scaffold under a tensile solicitation consisting of a single layer with 16 unit cells (G5, porosity = 80%, pore side length, *p* = 200 μm and fiber width, *d* = 80 μm).

**Figure 3:**
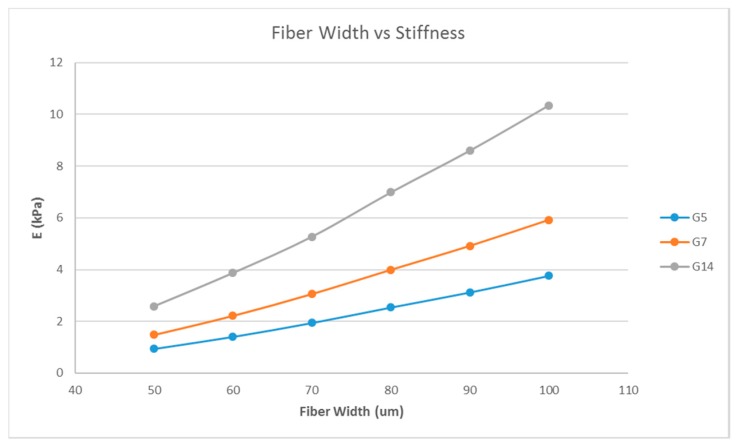
Effect of varying fiber widths on the stiffness of the structure consisting of a single layer with 16 unit cells with a fixed unit cell pore side length (*p* = 200 μm) for G5, G7, and G14.

A single-layer scaffold with 16 unit cells with the unit cell pore side length ‘*p*’ fixed at 200 μm was used; the fiber width ‘*d*’ was varied from 50 to 100 μm in steps of 10 μm and the simulation was performed. The ‘∆*L*’ value obtained from the simulation was used to calculate the stiffness of the structure. For example, the simulation results are shown in Figure 2 for one case; the maximum deformation value was taken as ‘∆*L*’. The effect of varying fiber width on the stiffness of the structure is shown as a plot in Figure 3.

### 4.2. Influence of the Porosity on the Stiffness of the Structure

**Figure 4:**
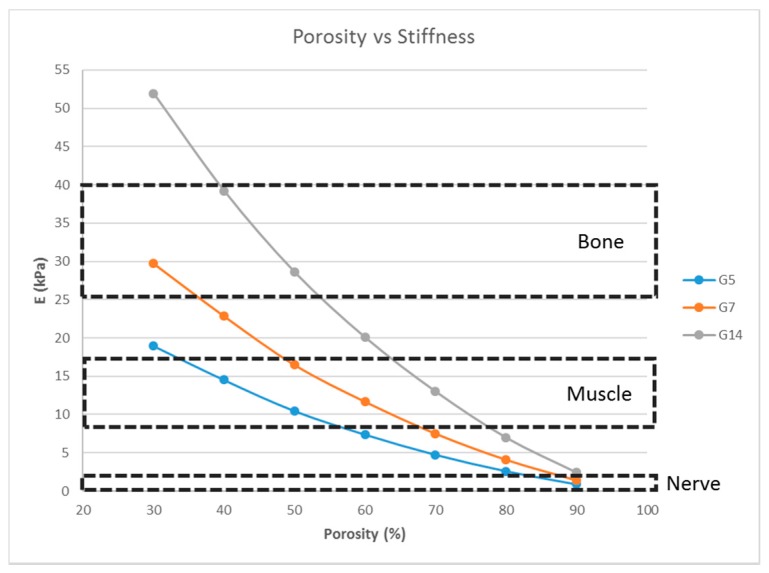
Effect of the porosity on the stiffness of the structure consisting of a single layer with 16 unit cells with a fixed fiber width (*d* = 100 μm) for G5, G7, and G14.

A single-layer scaffold with 16 unit cells with the fiber width ‘*d*’ fixed at 100 μm was used; the porosity (hence, the pore size) was varied from 30% to 90% in steps of 10% and the simulation was performed. A porosity range of 30% to 90% is commonly used for tissue engineering scaffolds [12,26,27] and, hence, the same range was used in this study. The effect of the variation of the porosity on the stiffness of the structure was determined (Figure 4). 

### 4.3. Effect of the Number of Unit Cells per Layer on the Stiffness of the Structure

**Figure 5:**
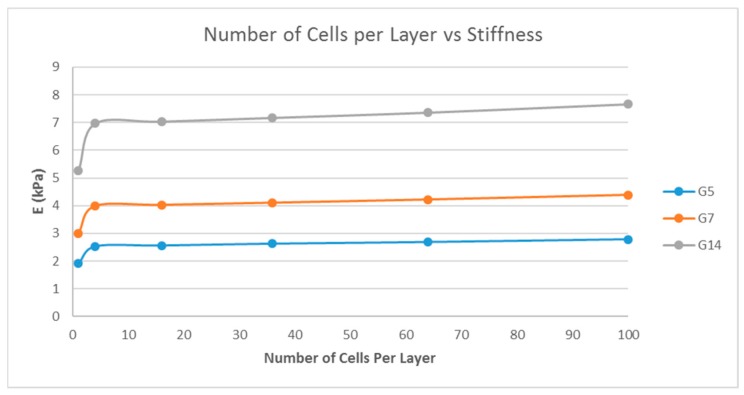
Effect of the number of unit cells per layer on the stiffness of the structure with a fixed fiber width (*d* = 80 μm), porosity (=80%), and unit cell pore side length (*p* = 200 μm) for G5, G7, and G14.

A single-layer scaffold with a variable number of cuboidal unit cells with the unit cell pore side length ‘*p*’ fixed at 200 μm, a fiber width of 80 μm, and a porosity of 80% was used; the number of unit cells per layer being varied as 1, 4, 16, 36, 64, and 100 unit cells and the simulation was performed. The pore side length used was 200 μm because pore sizes greater than 100 μm are required for macroporous scaffolds for better cell penetration and proliferation [26]. The effect of the number of unit cells per layer on the stiffness of the structure was determined and is shown in Figure 5.

### 4.4. Effect of the Number of Layers on the Stiffness of the Structure

**Figure 6:**
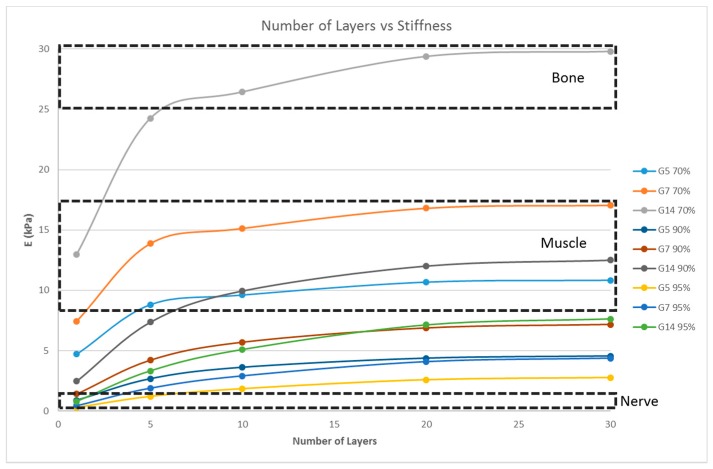
Effect of the number of layers on the stiffness of the structure with a fixed unit cell pore side length (*p* = 200 μm) at three different porosity values (70%, 90%, 95%) for G5, G7, and G14.

Scaffolds consist of multiple layers and, hence, the number of layers also plays an important role in determining the stiffness of the matrix. The influence of the number of layers on the scaffold stiffness is shown in Figure 6. A scaffold with 16 unit cells per layer, with the unit cell pore side length ‘*p*’ fixed at 200 μm, and at three porosity values of 70%, 90%, and 95% were considered; the number of layers being varied as 1, 5, 10, 20, and 30; and the simulation was performed.

### 4.5. Effect of the Material Selection on the Stiffness of the Structure

**Figure 7:**
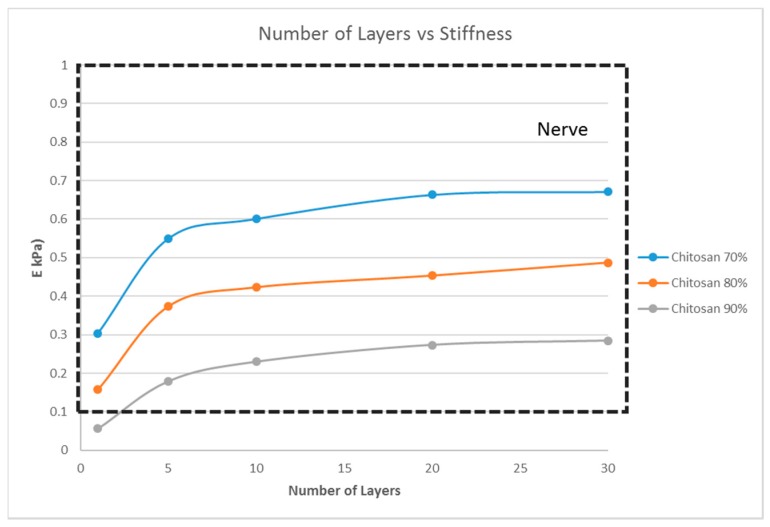
Effect of the number of layers on the stiffness of the structure with a fixed unit cell pore side length (*p* = 200 μm) at three different porosity values (70%, 80%, 90%) for chitosan.

**Figure 8:**
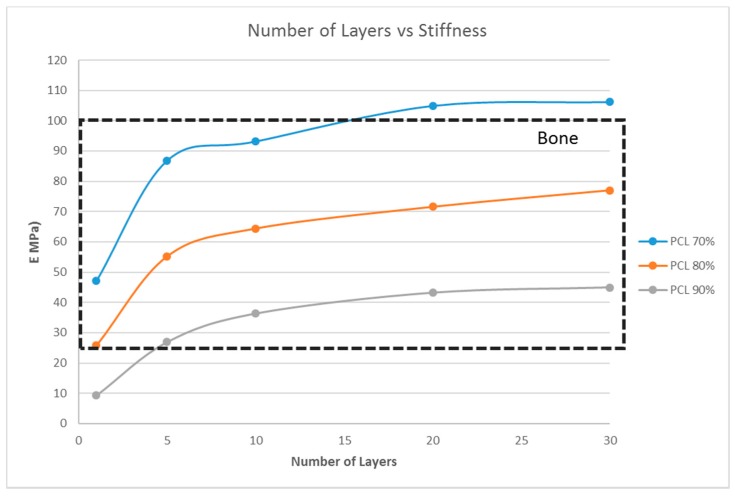
Effect of the number of layers on the stiffness of the structure with a fixed unit cell pore side length (*p* = 200 μm) at three different porosity values (70%, 80%, 90%) for PCL.

From the previous sections, with G5, G7, and G14 as the scaffold materials, scaffold designs with the stiffness range for myogenic and osteogenic lineages were obtained easily, while there are only limited scaffold designs with the stiffness range for the neurogenic lineage. The scaffold designs corresponding to the stiffness range suitable for the neurogenic lineage, as determined in the previous sections, were single-layer scaffolds with high porosity, which is practically difficult to fabricate and handle. This limitation could be overcome by choosing a different material. This section studies two other materials, namely PCL and chitosan, and its effects on the scaffold stiffness (Figure 7 and Figure 8).

## 5. Discussion

In an effort to elucidate the importance of scaffold design in directing the stem cell lineage differentiation, we studied five prominent parameters of porous tissue engineering scaffolds, namely, fiber width, porosity, number of unit cells per layer, number of layers, and material selection, and their influence on the matrix stiffness. By proper selection of these parameters, the stiffness of the structure can be tuned to the desired value in line with the intended stem cell lineage, neurogenic, myogenic, or osteogenic lineages. It is a well-established fact that matrix stiffness plays a major role in directing the stem cell lineage specification. In a seminal study, Engler et al. [7] demonstrated that mesenchymal stem cells can be stimulated to differentiate into neurons and osteoblasts when plated on soft and stiff matrices, respectively, that were chemically similar. In another study by Sharma et al. [28], substrates with mechanical property gradients were evaluated for their ability to direct bone marrow stromal cell differentiation along osteogenic and tenogenic lineages. Increased osteogenic fate was observed on fibronectin substrates and tenogenic differentiation fate was observed on collagen substrates, and these responses further depended on the stiffness of the substrate. From their study on the effect of matrix stiffness on the differentiation of bone marrow MSCs, Park et al. [10] concluded that a stiff matrix promotes MSC differentiation into a smooth muscle cell lineage, while a soft matrix (~1 kPa) promotes MSC differentiation into the chondrogenic and adipogenic lineages. However, almost all of the studies proving this claim were done on 2D substrates. There are very few studies done with 3D cultures. Pek et al. [29] have validated the effect of matrix stiffness on the differentiation of hMSCs in 3D, an effect that was reported previously for the culture of stem cells on a 2D substrate by using thixotropic gels for 3D cell culture. Such studies done on 3D matrices were mostly hydrogel substrates and the matrix stiffness depends only on the thickness of the hydrogel construct and the choice of hydrogels. The 3D biomimetic scaffolds play an important role in tissue engineering and it is important to study the effect of the design of such scaffolds on stem cell differentiation. The present study is one of the earliest attempts to evaluate the potential of designing porous scaffolds with tunable matrix stiffness for directing the stem cell lineage specification. 

Matrix stiffness can be varied by varying the fiber width. From Figure 3, the stiffness increases with increasing fiber width. A related parameter is the porosity of the scaffold structure. Figure 4 indicates that the stiffness of the structure decreases with increasing porosity. The findings are in line with our expectations and previous studies [19,27]. In addition, in Figure 4, it is shown that by varying the porosity of the scaffold, the stiffness can be tuned to various ranges. For instance, with G7 as the material, a porosity of 90% yields a stiffness range suitable for the neurogenic lineage, while 60% porosity results in a matrix stiffness for the myogenic lineage. The effect of varying the number of unit cells per layer on the matrix stiffness was studied and shown in Figure 5. The number of unit cells was increased from a single unit cell per layer to 100 unit cells per layer. It is seen that the matrix stiffness is least sensitive to the number of unit cells per layer. The stiffness does not significantly increase with a hundred-fold increase in the number of unit cells per layer. Hence, while designing the scaffolds for stem cell differentiation, the size of the scaffold, in terms of length and breadth (two-dimensional), can be chosen based on the ease of the user and intended assays, from a six-well plate to a 96-well plate, as this parameter does not affect the matrix stiffness to a great extent so as to influence the stem cell lineage specification. 

However, the thickness of the scaffold, as a measure of the number of layers, influences the matrix stiffness, as seen in Figure 6. For G14 material, a single layer scaffold of 16 unit cells with 70% porosity yields a stiffness range for the myogenic lineage, while multi-layer scaffolds of 10 layers and above with 16 unit cells in each layer with the same porosity of 70% has the stiffness range suitable for the osteogenic lineage. However, it is important to note that thicker scaffolds (>500 μm) might result in hypoxic gradients and, hence, affect the differentiating cell metabolism [30]. Though the effects of hypoxic gradients are less pronounced in macroporous scaffolds, this study also helps in determining the minimum number of layers for a particular material-pore size-porosity combination so that the thickness can be kept below 500 μm without compromising the matrix stiffness. For instance, in Figure 6, G14 70%, a scaffold with one layer, has a stiffness in the neurogenic range while 10 layers and above are in the osteogenic range. Hence, 10 layers will ensure that the stiffness is in the osteogenic range and there is no need to go up to 30 layers, avoiding much thicker scaffolds and hypoxic gradients. Lastly, the effect of material selection is also studied. If chitosan is used as the material for scaffold fabrication, irrespective of the number of layers and porosity, the stiffness range always lies within the neurogenic lineage (<1 kPa) as shown in Figure 7. Similarly, if the material considered is PCL, then the range of stiffness obtained is always in the osteogenic lineage range, irrespective of the number of layers or the porosity.

This study offers a good guideline for design of porous tissue engineering scaffolds with tunable matrix stiffness for directing stem cell lineage specification. By optimally selecting the fiber width, porosity, the number of layers and material, the matrix stiffness of the desired range can be obtained. For applications which require a specific porosity, the porosity can be fixed at the desired value and the other parameters, namely the number of layers or material selection, can be varied to obtain the scaffolds with desired stiffness for directing the stem cell differentiation towards a particular lineage. Similarly, if there is a constraint on the usage of materials due to the nature of the fabrication process, the design parameters, like fiber width, porosity, and number of layers, can be varied. Thus, the study offers a guideline for selection of scaffold design for stem cell lineage specification. The present work considered the simplest and most common unit cell design, namely a cubic structure. In the future, other complex unit cell structures could be designed and the effect of such unit cell structures on the matrix stiffness could be studied.

## 6. Challenges

There are certain challenges that need to be addressed by further advanced research. The first challenge is the printing of porous structures of various materials for experimental validation. Not all materials are easily printable into 3D porous scaffolds for experimental validation. While PCL is a widely-used biomaterial for fabrication of 3D scaffolds [31,32] and can be easily printed into 3D scaffolds using various 3D printing methods, optimization of parameters (nozzle diameter, flow rate, solution concentration, cross-linking) are required for printing of hydrogels made of gelatin and chitosan to fabricate 3D scaffolds of desired pore size and fiber diameter. The limitations of the fabricating method (solution viscosity, resolution, accuracy) should also be taken into account. The second challenge is the experimental validation of the simulation results. The choice of materials not only affects the matrix stiffness but also plays a crucial role in the regulation of biochemical and ECM cues, such as integrin-ligand binding. These regulatory aspects, in turn, determine the differentiation of stem cells into various lineages. Hence, it is important to note that even if scaffolds with a desired matrix stiffness (based on the intended application) are fabricated by varying the scaffold parameters and an appropriate choice of material, stem cells might differentiate into a different lineage, far beyond the expectation, because of the biochemical and ECM cues which are material-dependent. In the present study, a cubic unit cell structure is adopted for simplicity. Using a different unit cell geometry, like hexagonal or orthogonal unit cells, will alter the mechanical properties of the scaffold structure and, in turn, influence the differentiation of stem cells [22]. Another challenge is the determination of structural failure modes for each scaffold design and material. A detailed understanding and application of the in vivo loading conditions is a prerequisite to assess the failure mechanisms of the scaffolds. In addition to the scaffold design, optimization of process parameters to ensure proper printing quality also plays a role in determining the failure modes of the scaffolds as poor printing quality might result in high localized stresses, thereby affecting the failure mechanism. Lastly, the challenge presented by the effect of degradation of the biomaterial on the mechanical properties of the scaffold should be considered. The matrix stiffness decreases as the material undergoes degradation. Further experimental studies are required to establish the effect of degradation on matrix stiffness and, in turn, on the stem cell differentiation, which is difficult to consider in numerical simulations.

## 7. Conclusions

A new approach of designing 3D porous tissue engineering scaffolds with tunable stiffness for directing stem cell lineage specification is studied. The effect of various scaffold parameters, namely fiber width, porosity, number of unit cells per layer, number of layers and material selection on the matrix stiffness is studied. Matrix stiffness increases with increasing fiber width and decreases with increasing porosity. The number of unit cells per layer does not significantly influence the stiffness of the structure. The thickness of the scaffold as a measure of number of layers influences the stiffness of the structure significantly. Selection of materials is also an important criterion for achieving the desired stiffness. In the present study, irrespective of the scaffold design (porosity and number of layers), chitosan always yields a stiffness range suitable for the neurogenic lineage while PCL gives a stiffness range for the osteogenic lineage. The study, thus, provides a good guideline for the design of porous tissue engineering scaffolds with tunable matrix stiffness for directing stem cell lineage specifications.
